# Association between metabolic syndrome and salivary MMP‐8, myeloperoxidase in periodontitis

**DOI:** 10.1111/odi.15014

**Published:** 2024-06-09

**Authors:** Julie Toby Thomas, Betsy Joseph, Sajit Varghese, Nebu George Thomas, Baiju Kamalasanan Vijayakumary, Timo Sorsa, Sukumaran Anil, Tuomas Waltimo

**Affiliations:** ^1^ Department of Oral and Maxillofacial Diseases University of Helsinki and Helsinki University Hospital Helsinki Finland; ^2^ Department of Periodontics, Saveetha Dental College and Hospitals Saveetha Institute of Medical and Technical Sciences Chennai Tamilnadu India; ^3^ Department of General Medicine Pushpagiri Institute of Medical Sciences and Research Centre Thiruvalla Kerala India; ^4^ Department of Periodontics Pushpagiri College of Dental Sciences Thiruvalla Kerala India; ^5^ Department of Statistics Women's College Trivandrum Kerala India; ^6^ Division of Periodontology, Department of Dental Medicine Karolinska Institutet Stockholm Sweden; ^7^ Department of Dentistry Hamad Medical Corporation Doha Qatar; ^8^ College of Dental Medicine Qatar University Doha Qatar; ^9^ Department of Oral Health and Medicine, University Center for Dental Medicine Basel University of Basel Basel Switzerland

**Keywords:** ELISA, matrix metalloproteinase 8, metabolic syndrome, myeloperoxidase, periodontal disease, saliva, triglycerides

## Abstract

**Objective:**

This study investigated the effect of metabolic syndrome (MetS) on periodontal clinical parameters and salivary biomarkers' matrix metalloproteinase‐8 (MMP‐8) and myeloperoxidase (MPO) in patients with periodontitis.

**Methods:**

A total of 120 participants aged 25–55 were categorized into three groups: MetS with periodontitis (*n* = 40); systemically healthy with periodontitis (*n* = 40); and systemically and periodontally healthy controls (*n* = 40). Data collected included systemic parameters like waist circumference (WC), blood pressure (BP), high‐ and low‐density lipoproteins, triglycerides (TG), fasting blood sugar (FBS), and glycated hemoglobin (HbA1c). Periodontal parameters estimated included bleeding on probing score (BoP), full‐mouth plaque score (FMPS), periodontal probing depth (PPD), clinical attachment loss (CAL), and the number of missing teeth. Unstimulated whole saliva was analyzed via ELISA for active MMP‐8 (aMMP‐8), total MMP‐8 (tMMP‐8), and MPO.

**Results:**

Participants with MetS and periodontitis exhibited significantly higher periodontal parameters, salivary aMMP‐8, and MPO (26.26 vs. 24.1 ng/mL and 13.53 vs. 11.55 ng/mL compared to systemically healthy periodontitis patients) (all *p* < 0.01). Positive correlations occurred between aMMP‐8 and WC, TG, and FBS (*p* < 0.01), and between MPO and WC, BP, and TG (*p* < 0.01).

**Conclusions:**

The positive associations between these biomarkers and metabolic parameters indicate their potential utility for monitoring cardiovascular and glycemic risk in patients with periodontal disease.

## 1 INTRODUCTION

Periodontitis is a chronic inflammatory condition driven by dysbiotic dental biofilms and is influenced by various environmental, genetic, and systemic factors, leading to the progressive destruction of tooth‐supporting tissues and attachment/bone loss (Tonetti et al., 2017). About 19% of adults worldwide suffer from periodontal disease, and its incidence is continually rising (World Health Organization, 2022). Periodontal disease is intricately linked with several systemic conditions, including diabetes, cardiovascular disease, hormonal changes, obesity, hematologic and neurological disorders, and immune dysfunction, which can exacerbate inflammatory responses to pathogens (Jepsen et al., 2018).

Metabolic syndrome (MetS), characterized by increased abdominal obesity, elevated blood pressure, blood sugar dysregulation, high triglycerides (TG), and low‐density lipoprotein (LDL), is increasingly found to be predominant among Indian adults, posing heightened risks of diabetes and cardiovascular disease (Sundarakumar et al., 2022). Both periodontal disease and metabolic syndrome trigger proinflammatory and immune responses, sharing common mediators such as C‐reactive protein, prostaglandin E2, interleukin‐1β, and tumor necrosis factor‐alpha. Therefore, understanding the potential associations between MetS and periodontal disease is crucial (Pirih et al., 2021).

Research has demonstrated the adverse effects of metabolic syndrome on periodontal clinical parameters, including increased tooth loss, deeper periodontal pockets, greater clinical attachment loss, heightened alveolar bone loss, and enhanced tooth mobility (Kotin et al., 2022). Moreover, a positive association between metabolic syndrome and periodontal disease has been confirmed in a systematic review by Gobin et al. (2020).

Matrix metalloproteinase‐8 (MMP‐8) and myeloperoxidase (MPO) are key biomarkers involved in the inflammatory cascade of periodontal disease. Total (tMMP‐8), in particular, exists in both latent and active forms, with active MMP‐8 (aMMP‐8) serving as a reliable marker for subclinical periodontal destruction (Räisänen et al., 2023). Sorsa et al. (2021) demonstrated that aMMP8 level > 20 ng/mL reflects active proinflammatory, tissue‐destructive processes in periodontitis. Furthermore, Han, Shin, et al. (2012) reported increased expression of MMP‐8, MMP‐9, and MMP‐13 in GCF (gingival crevicular fluid) of patients with metabolic syndrome. Endotoxemia due to increased circulating lipopolysaccharide has been found to contribute to the pathogenesis of cardiometabolic disorder (Pussinen et al., 2022). Promising findings were obtained when the aMMP‐8 chair‐side test was used to evaluate the inflammatory status very early stages of periodontal disease. In addition to traditional clinical approaches, the aMMP‐8 chair‐side test may be a useful supplementary diagnostic tool for the detection of periodontal disease (Leppilahti et al., 2011).

Myeloperoxidase, on the other hand, plays a critical role in inflammation and oxidative stress. The neutrophil‐expressed enzyme inducing latent MMP activation and reactive oxygen species production was found to be upregulated in periodontal tissues, aggravating the inflammatory condition. Irrespective of periodontitis, a recent systematic review by Nijakowski et al. (2023) reported that elevated MPO in saliva and serum impacts cardiovascular disease pathogenesis. Furthermore, research should investigate salivary MPO's potential as a cardiovascular risk predictor in periodontitis.

While prior evidence links metabolic syndrome and clinical periodontal parameters, we aimed to advance etiopathogenic understanding by incorporating proinflammatory oral fluid biomarkers aMMP‐8, tMMP‐8, and MPO alongside clinical parameters. Therefore, we hypothesize that periodontitis patients with metabolic syndrome will exhibit higher levels of salivary aMMP‐8, tMMP‐8, and MPO compared to systemically healthy patients with periodontitis. This study aimed to evaluate and compare the salivary expression of aMMP‐8, tMMP‐8, and MPO among periodontitis patients with and without metabolic syndrome. We also aimed to investigate the correlation of these salivary biomarkers with periodontal and systemic parameters.

## 2 MATERIALS AND METHODS

### 2.1 Study design

This cross‐sectional study was conducted at the Pushpagiri Institute of Medical Sciences and Research Centre, Kerala, India, from June 15, 2023, to December 15, 2023. The Pushpagiri Institute of Medical Sciences and Research Center Institutional Review Board approved this study on May 5, 2023 (IRB Study Reference No: 20/0112023). All procedures were conducted by the revised version of the Declaration of Helsinki. The study is reported based on the Strengthening the Reporting of Observational Studies in Epidemiology (STROBE) guidelines.

### 2.2 Participants

The study participants comprised both male and female Indian adults aged between 25 and 55 years. The sample size (*n*) was calculated using the formula:

$$n=\frac{2{\left(\text{Standard deviation}\right)}^{2}}{{\left(\text{Effect size}\right)}^{2}}{\left(Z_{\alpha /2}+Z_{1-\beta }\right)}^{2}$$

where *Z*_*α*/2_ = 1.96 and *Z*_1−*β*_ = 1.28 are the 95% confidence values obtained from a standard normal distribution, at least 40 subjects were required to detect a moderate difference in salivary biomarkers, with an effect size of 3.00 and a standard deviation of 4.14 ng/mL from a pilot survey. Hence, the final minimum sample size required was 40 participants per group.

Using systematic random sampling, a sample of 120 participants was selected from patients at the Department of Periodontology and Dental Outpatient Department at Pushpagiri Institute of Medical Sciences and Research Center, Kerala. Before recruitment, the primary investigator (JTT) executed basic periodontal screening with a World Health Organization (WHO) probe on all patients (Claydon et al., 2022). Participants were assigned a score of 4, indicating probing depth >5.5 mm in at least two sextants, and underwent detailed periodontal examinations by two investigators (JTT and BJ). A sampling interval of 5 was calculated based on the estimated patient volume. Every 5th patient diagnosed with stage 3 or stage 4 periodontitis and had at least 20 teeth was selected to participate. Periodontitis case categorization was based on ≥5 mm clinical attachment loss (CAL) and ≥6 mm probing pocket depth (PPD) in more than 30% of sites with radiographic bone loss up to the middle third of the root under the 2018 EFP/AAP periodontitis case classification (Ortigara et al., 2021). Bleeding on probing (BoP) <10% of sites, PPD ≤3 mm, and no CAL indicated periodontal health (Caton et al., 2018).

Based on the presence or absence of metabolic syndrome, the included participants were segregated into two groups. Sampling continued until the desired sample size of 40 participants was met in each group. The diagnostic criteria for identifying metabolic syndrome were based on the 2006 International Diabetes Federation (IDF) guidelines (Rezaianzadeh et al., 2012). Specifically, the IDF criteria for metabolic syndrome include: (a) increased waist circumference (WC), with cutoff levels based on Asian populations of ≥90 cm for men and ≥80 cm for women; and (b) the presence of any two of the following four factors: (1) triglycerides (TG) ≥150 mg/dL or specific treatment for this lipid abnormality; (2) reduced high‐density lipoprotein (HDL) <40 mg/dL in men and <50 mg/dL in women or specific treatment; (3) raised blood pressure, systolic BP ≥130 or diastolic BP ≥85 mm Hg or treatment for previously diagnosed hypertension; and (4) raised fasting plasma glucose (FPG) ≥100 mg/dL (5.6 mmol/L) or previously diagnosed type 2 diabetes (T2DM) (Alberti et al., 2006).

**FIGURE 1:**
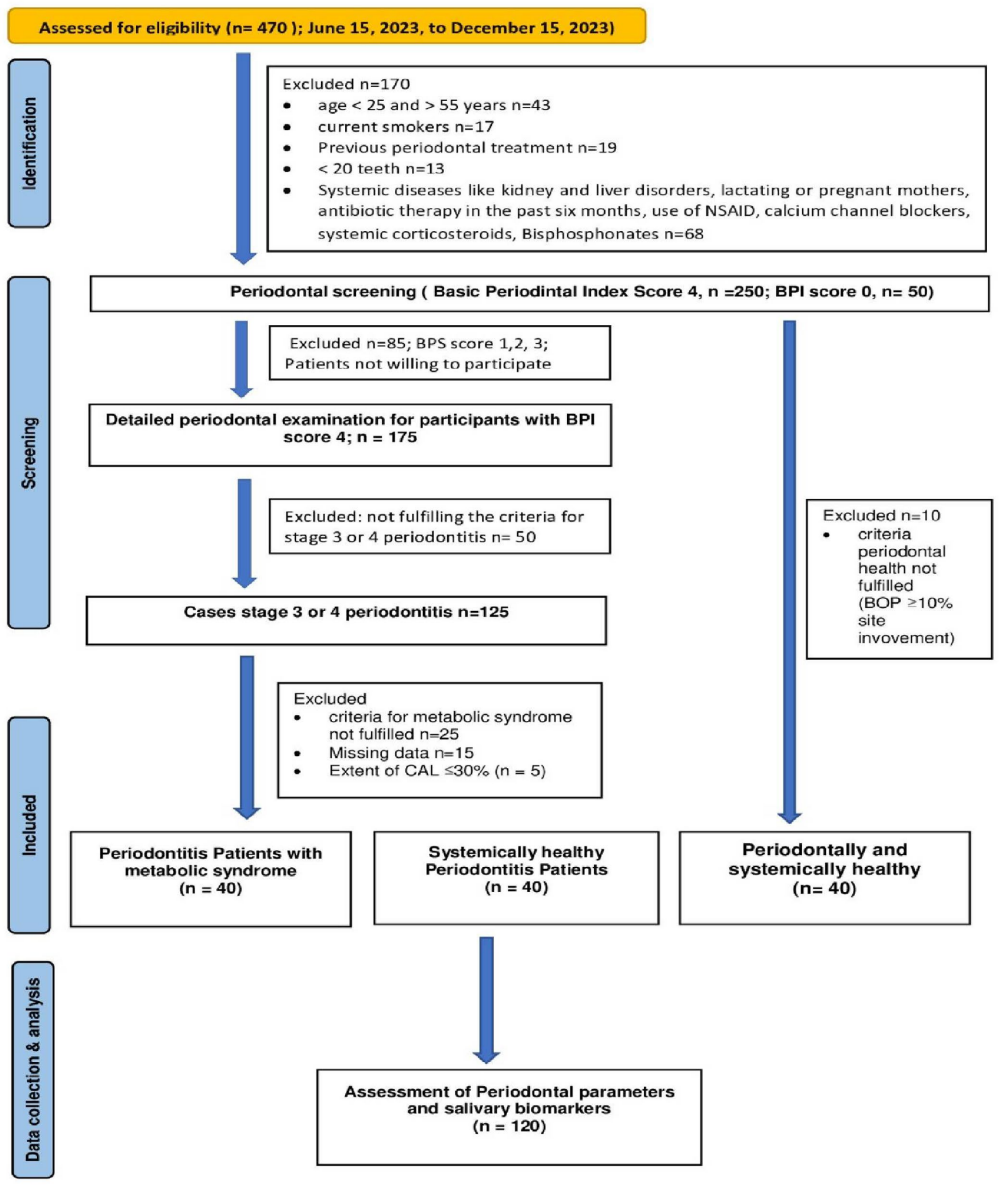
STROBE diagram on patient recruitment.

Forty systemically and periodontally healthy participants registered for restorative dental procedures or reported for routine dental check‐ups were recruited as the control group from the Dental Outpatient Department at Pushpagiri Institute of Medical Sciences and Research Center, Kerala. Participants were excluded if they reported a history of any systemic diseases or conditions other than metabolic syndrome, had previous periodontal treatment or antibiotic therapy in the past 6 months, had less than 20 teeth, were on bisphosphonate therapy, were current smokers, and were on nonsteroidal anti‐inflammatory drugs or topical/systemic corticosteroids, and calcium channel blockers, insulin, those who were pregnant or lactating mothers, or did not provide informed consent (Figure 1).

The study participants were categorized into three groups:

Group 1: 40 participants with metabolic syndrome diagnosed with periodontitis (MetS‐PD).
Group 2: 40 systemically healthy participants diagnosed with periodontitis (SH‐PD).
Group 3: 40 systemically healthy participants with healthy periodontium (SH‐PH).

### 2.3 Variables

#### 2.3.1 Sociodemographic details and oral hygiene practices

A self‐validated closed‐ended patient‐reported questionnaire was used to gather details on the place of residence, age, gender, education level, medical history, smoking habits, previous dental history, and routine oral hygiene practices before initiating sample collection (Appendix: Sociodemographic and Oral Hygiene Questionnaire).

#### 2.3.2 Assessment of anthropometric parameters

A third investigator (SJ) conducted the physical examination of all consenting participants and recorded measurements, including systolic and diastolic blood pressure, body weight in kg, height in meters, and waist circumference. Body mass index (BMI) in kg/m^2^ was calculated by dividing body weight (kg) by the square of height (m). Weight was measured in kilograms using a mechanical scale (Prestige, HM007, India), and height was determined using a stadiometer. The BMI of study participants was recorded. Waist circumference (WC) was measured in cm at the umbilical level using a measuring tape (PWT80G—Perfect Waist & Body Tape Measure, Ohio), with the subject standing with feet 20–30 cm apart. After 5–10 min of rest, blood pressure was measured from the right arm using a digital sphygmomanometer (Omron HEM 7120, Vietnam), with subjects seated with their backs rested on a chair. The average of two blood pressure readings was recorded.

#### 2.3.3 Assessment of serum biochemical parameters

After 12–14 h of fasting, blood samples were collected by investigator SJ by the standardized protocol from all consenting participants for the estimation of triglycerides TG, LDL, HDL, FBG, and glycated hemoglobin levels (HbA1c) (Tuck et al., 2009).

#### 2.3.4 Clinical periodontal parameters

Periodontal parameters were assessed by two blinded investigators (JTT and BJ). Both the examiners underwent training by a specialist in periodontology (SA) before interexaminer calibration and clinical examination. Calibrations were performed for all the periodontal indices on 10 participants at different time intervals. The interexaminer variability was analyzed, and a Cohen Kappa score ≥0.80 was considered to be acceptable.

The recorded periodontal parameters for each participant included the percentage of sites with BoP (Trombelli et al., 2018), PPD, CAL, gingival recession (GR), number of missing teeth due to periodontitis, and full‐mouth plaque score (FMPS) calculated as the percentage of sites with plaque (D'Elia et al., 2023).

Measurements were taken at six sites per tooth (mesio‐buccal, mesio‐lingual, mid‐buccal, disto‐buccal, disto‐lingual, and mid‐lingual) using a UNC‐15 probe (Hu‐Freidy's, USA). BoP was recorded dichotomously as present or absent within 10 s of probing each site. PPD was measured from the free gingival margin to the base of the pocket. CAL was measured from the cementoenamel junction to the bottom of the pocket or sulcus (Armitage, 2004). GR was measured along the tooth axis as the distance from the CEJ to the gingival margin.

#### 2.3.5 Biochemical analysis of active MMP‐8

##### Collection of saliva

The study participants were advised to abstain from food, fluids, and chewing gum for 1 h before saliva collection and to report between 10 and 12 AM. Sample collection was performed by investigators blinded to patient group allocation. Unstimulated saliva (3 mL) was collected from each participant by the spitting method, following published guidelines (Navazesh & Kumar, 2008). The patients were seated in an upright position for 5 min on a dental chair and advised to expectorate every 60 s into a funnel connected to 5‐mL sterile falcon tubes (Maxome Labsciences, Bengaluru, India). Saliva samples were stored at −20°C until analysis.

##### Qualitative estimation of active MMP‐8

A commercial lateral flow mouth rinse immunoassay test (PerioSafe, Dentognostics GmbH) was used for qualitative analysis of active MMP‐8 (aMMP‐8) in oral rinse samples (Sorsa et al., 2021). Testing was performed by a single trained and calibrated investigator (JTT). Participants were pre‐rinsed with tap water for 30 s before sample collection. For sample collection, participants rinsed with 5 mL sterile distilled water for 30 s and expectorated into a collection cup. This lateral flow immunoassay detects aMMP‐8 fragments from 20 to 35 kDa. One to 3 mL of oral rinse was aspirated using a 3‐mL syringe fitted with a 0.45‐μm filter, and 3–4 drops of filtered sample were added to the test system. After 5 min, negative aMMP‐8 results showed one test line, while two test lines indicated sample positivity, suggesting higher periodontitis risk (Leppilahti et al., 2011).

##### Quantitative estimation of salivary biomarkers using human active MMP‐8, total MMP‐8, and MPO ELISA kit

Saliva samples stored at −20°C were subjected to quantitative sandwich ELISA analysis for aMMP‐8, total MMP‐8 (tMMP‐8), and MPO according to the manufacturer's guidelines. The stored saliva was thawed and centrifuged at 1000 × *g* at 2–8°C for 15 min to remove particulates; the clear supernatant was collected to estimate the markers using commercially procured ELISA kits for Human total MMP‐8 (Catalog No: KGH0903), Human active MMP‐8 (Catalog No: EK0464; Boster Bio), and Human MPO ELISA Kit (Catalog No: KBH0880) (KRISHGEN Biosystems). Color change of enzyme‐substrate reactions was measured spectrophotometrically at a wavelength of 450 nm. The limit of detection levels for the tests using the ELISA kits for active and total MMP‐8 (Human MMP‐8 activity assay) as well as MPO (Human MPO assay) was 28, 30, and 0.7 ng/mL, respectively.

### 2.4 Statistical analysis

Statistical analyses were performed using IBM SPSS version 26.0. Descriptive and categorical variables were summarized as frequencies and percentages. Continuous variables were summarized as means and standard deviation. Based on the statistical central limit theorem, parametric tests were conducted for each group since the sample size was above 30. One‐way ANOVA was used to analyze group differences, with post hoc tests for inter‐group comparisons. A *p*‐value <0.05 was considered statistically significant. Pearson correlation coefficients (*r*) were calculated to determine correlations between periodontal variables, metabolic syndrome‐related systemic parameters, and salivary biochemical measures of aMMP‐8, tMMP‐8, and MPO.

## 3 RESULTS

### 3.1 Subject characteristics

One hundred and twenty participants were categorized into three groups: MetS‐PD (*n* = 40), SH‐PD (*n* = 40), and SH‐PH (*n* = 40). Mean participant age in years in groups MetS‐PD, SH‐PD, and SH‐PH was 45.35 ± 8.74, 47.25 ± 5.11, and 43.4 ± 8.78. The difference in age among the groups was not statistically significant (*p* > 0.05).

Despite the varied proportion of males and females between groups, the distribution was not statistically significant (*p* = 0.222). Place of residence (rural vs. urban) and education level distributions were also homogeneous between groups (*p* = 0.905 and *p* = 0.604, respectively) (Table 1).

**TABLE 1:** Demographic characteristics of participants according to the different groups.

Variables		MetS‐PD (*N* = 40)	SH‐PD (*N* = 40)	SH‐PH (*N* = 40)	*p*‐Value
Age (years)	Mean ± SD	45.35 ± 8.74	47.25 ± 5.11	43.4 ± 8.78	0.116
Gender	Male, *n* (%)	13 (32.5)	7 (17.5)	13 (32.5)	0.222
	Female, *n* (%)	27 (67.5)	33 (82.5)	27 (67.5)	
Place of residence	Rural, *n* (%)	20 (50.0)	19 (47.5)	21 (52.5)	0.905
	Urban, *n* (%)	20 (50)	21 (52.5)	19 (47.5)	
Educational Qualification	Graduate/Postgraduate, *n* (%)	15 (37.5)	9 (22.5)	12 (30)	0.604
	Completed High school, *n* (%)	19 (47.5)	23 (57.5)	23 (57.5)	
	Completed primary school/illiterate, *n* (%)	6 (15)	8 (20)	5 (12.5)	

*Note*: Values are expressed as mean ± standard deviation and number (percentage). One‐way analysis of variance (ANOVA) followed by post hoc Tukey test comparisons was used for continuous variables. *p‐*Values were tested using Pearson's chi‐square test for categorical variables.

Patient‐reported oral health problems differed significantly between groups (Table 2). Behaviors related to oral hygiene, including brushing frequency, cleaning method, and dental visit frequency, were homogeneous among groups (*p* = 0.52, *p* = 0.67, *p* = 0.549). Reports of halitosis and bleeding on brushing differed significantly between groups (*p* < 0.01), occurring more frequently in MetS‐PD. The frequency of positive chair‐side aMMP‐8 tests also differed significantly (*p* < 0.01), with 90% of MetS‐PD and 85% of SH‐PD testing positive versus 0% in SH‐PH. Patients with a known history of diabetes, dyslipidemia, or hypertension were on oral medications like metformin (*n* = 22), atorvastatin (*n* = 15), and telmisartan (*n* = 8).

**TABLE 2:** Information related to oral health habits and problems, and aMMP‐8 POC kit.

Variables		MetS‐PD (*N* = 40)	SH‐PD (*N* = 40)	SH‐PH (*N* = 40)	*p‐*Value
Frequency of brushing	Once a day	26 (65)	28 (70)	31 (77.5)	0.52
	Twice a day	14 (35)	12 (30)	9 (22.5)	
Method of cleaning teeth	Use only toothbrush	1 (2.5)	0 (0)	0 (0)	0.67
	Toothbrush and paste	36 (90)	38 (95)	38 (95)	
	Use of charcoal power	3 (7.5)	2 (5)	2 (5)	
Complaint of halitosis	Often	6 (15)	2 (5)	0 (0)	<0.01**
	Sometimes	21 (52.5)	4 (10)	8 (20)	
	Occasional/never	12 (32.5)	34 (85)	32 (80)	
Bleeding on brushing	Often	3 (7.5)	1 (2.5)	0 (0)	<0.01**
	Sometimes	23 (57.5)	6 (15)	14 (35)	
	Occasional/never	14 (35)	33 (82.5)	26 (65)	
Frequency of dental visit	Once in a year	2 (5)	4 (10)	2 (5)	0.549
	Occasional	37 (92.5)	36 (90)	38 (95)	
	Never	1 (2.5)	0 (0)	0 (0)	
aMMP‐8 POC kit	Negative	4 (10)	6 (15)	40 (100)	<0.01**
	Positive	36 (90)	34 (85)	0 (0)	

*Note*: **Statistically significant at 1% level (*p* < 0.01).

Abbreviation: aMMP‐8 POC, active‐matrix metalloproteinase‐8 point‐of‐care.

### 3.2 Comparison of periodontal, systemic, and salivary parameters

Using ANOVA, Table 3 compares systemic and periodontal parameters among MetS‐PD, SH‐PD, and SH‐PH groups. Irrespective of height, significant variation in weight and BMI was observed between groups (*p* < 0.01). Mean weight was higher in the MetS‐PD group (73.93 ± 10.09 kg) compared to SH‐PD (64.00 ± 9.70 kg) and SH‐PH (65.16 ± 10.20 kg). Similarly, BMI was significantly higher in the MetS‐PD group (30.59 ± 3.13 kg/m^2^) versus SH‐PD (27.32 ± 3.47 kg/m^2^) and SH‐PH (25.23 ± 4.18 kg/m^2^).

**TABLE 3:** Comparison of systemic and periodontal characteristics between groups.

Variables	MetS‐PD (*N* = 40)		SH‐PD (*N* = 40)		SH‐PH (*N* = 40)		*p*‐Value
	Mean	SD	Mean	SD	Mean	SD	
Height (mts)	1.56^a^	0.12	1.60^a^	0.22	1.61^a^	0.14	0.316
Weight (kg)	73.93^a^	10.09	64.00^b^	9.70	65.16^b^	10.20	<0.01**
BMI (kg/m^2^)	30.59^a^	3.13	27.32^b^	3.47	25.23^c^	4.18	<0.01**
WC (cm)	107.10^a^	11.13	97.14^b^	9.44	91.88^c^	13.67	<0.01**
SBP (mm of Hg)	130.00^a^	15.94	114.55^b^	10.50	116.75^b^	11.18	<0.01**
DBP (mm of Hg)	85.98^a^	10.35	76.90^b^	7.45	78.15^b^	5.60	<0.01**
TG mg/dL	133.70^a^	30.47	102.60^b^	30.92	97.18^b^	34.43	<0.01**
LDL (mg/dL)	140.73^a^	52.32	120.95^b^	30.79	89.85^c^	18.05	<0.01**
HDL (mg/dL)	57.86^a^	10.66	56.75^a^	8.60	54.93^a^	15.90	0.551
FBS (mg/dL)	148.38^a^	37.29	102.10^b^	12.12	97.05^b^	28.05	<0.01**
HbA1c (%)	6.88^a^	1.43	5.36^b^	0.61	5.31^b^	1.46	<0.01**
BoP (%)	56.68^a^	25.41	45.45^b^	27.53	7.99^c^	1.72	<0.01**
FMPS (%)	39.18^a^	16.75	38.40^a^	21.14	23.05^b^	12.78	<0.01**
PPD (mm)	6.70^a^	1.42	5.80^b^	1.22	3.03^c^	0.53	<0.01**
GR (mm)	2.33^a^	1.27	1.80^b^	1.05	0.37^c^	0.63	<0.01**
CAL (mm)	8.60^a^	2.04	7.63^b^	1.86	0.75^c^	1.32	<0.01**
Number of missing teeth	4.05^a^	2.60	2.68^b^	2.56	0.15^c^	0.66	<0.01**

*Note*: **Statistically significant at 1% level (*p* < 0.01); one‐way ANOVA test was used for continuous variables to analyze the differences across the three groups, and Scheffe's post hoc test was used for pairwise difference between the groups. Values with different superscripted letters indicate a statistically significant pairwise difference (*p* < 0.05) by Scheffe's post hoc test. Values with the same superscripted letters indicate a statistically significant pairwise difference (*p* > 0.05).

Abbreviations: BMI, Body mass index; BoP, Bleeding on probing; CAL, Clinical attachment loss; DBP, Diastolic blood pressure; FBS, Fasting blood sugar; FMPS, Full‐mouth plaque score; GR, Gingival recession; HbA1c, Glycated hemoglobin; HDL, High‐density lipoprotein; LDL, Low‐density lipoprotein; PPD, Probing pocket depth; SBP, Systolic blood pressure; TG, Triglycerides; WC, Waist circumference.

WC differed notably (*p* < 0.01), with higher means observed in MetS‐PD (107.10 ± 11.13 cm) compared to SH‐PD (97.14 ± 9.44 cm) and SH‐PH (91.88 ± 13.67 cm). Significant differences were also noted in systolic BP, diastolic BP, TG, LDL, FBS, and HbA1c (all *p* < 0.01). MetS‐PD exhibited higher means for these systemic biomarkers than the other groups. HDL levels did not differ significantly between groups (*p* = 0.551).

All periodontal parameters differed significantly between groups (*p* < 0.01), including BoP, FMPS, PPD, GR, CAL, and missing teeth. Means were higher in MetS‐PD compared to SH‐PD and SH‐PH across these variables.

**FIGURE 2:**
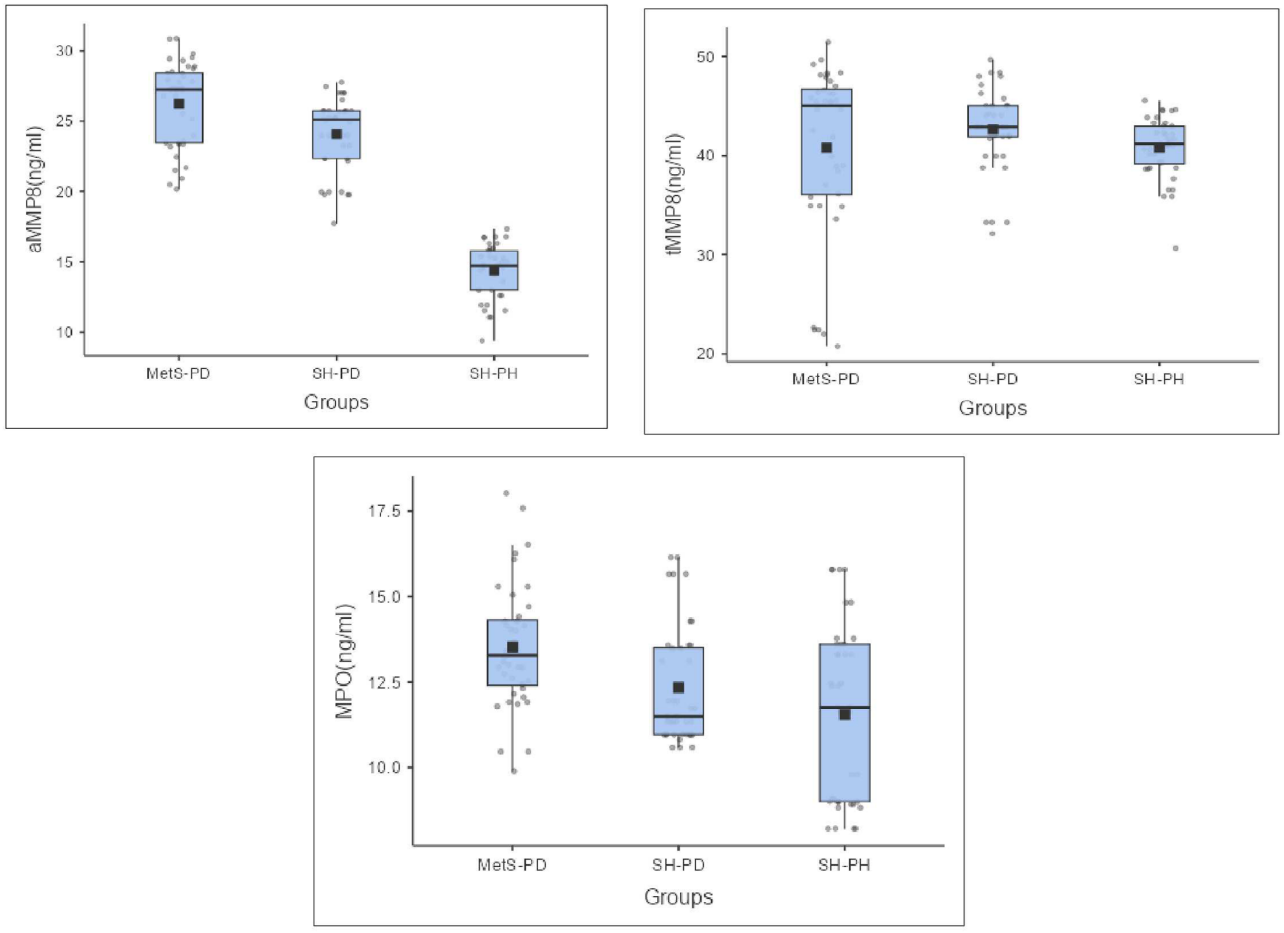
Comparison of salivary biomarkers in active metalloproteinase 8 (aMMP‐8), total MMP‐8 (tMMP‐8), and myeloperoxidase (MPO).

A comparison of salivary aMMP‐8, tMMP‐8, and MPO among the groups is illustrated in Figure 2. Salivary aMMP‐8 differed significantly between groups (*p* < 0.01), with a higher mean aMMP‐8 level in MetS‐PD (26.26 ± 3 ng/mL) compared to SH‐PD (24.1 ± 2.56 ng/mL) and SH‐PH (14.366 ± 1.89 ng/mL). In contrast, tMMP‐8 levels did not differ significantly (*p* = 0.269), with means of 40.80 ± 8.62 ng/mL (MetS‐PD), 42.63 ± 4.16 ng/mL (SH‐PD), and 40.81 ± 3.00 ng/mL (SH‐PH). Salivary MPO also exhibited significant between‐group differences (*p* < 0.01), with elevated levels in MetS‐PD (13.53 ± 1.80 ng/mL) versus SH‐PD (12.33 ± 1.72 ng/mL) and SH‐PH (11.55 ± 2.56 ng/mL).

### 3.3 Correlation of systemic parameters with periodontal parameters

WC exhibited significant moderate positive correlations with all periodontal parameters (all *p* < 0.01) except a weaker but still significant correlation with FMPS (*p* < 0.05, *r* = 0.180). BP (both systolic and diastolic) also showed substantial moderate positive correlations with BoP, PPD, GR, CAL, and missing teeth (*p* < 0.01), but not with plaque score.

TG demonstrated significant moderate positive correlations with BoP, FMPS, PPD, GR, and CAL (*p* < 0.01), but the association with missing teeth was non‐significant. Similar correlations were seen between LDL and periodontal parameters (*p* < 0.01). FBS significantly and moderately correlated with BoP, PPD, GR, CAL, and missing teeth (*p* < 0.01) but not with FMPS. HDL levels did not significantly correlate with most periodontal parameters, except for weak negative correlations with FMPS and GR.

Salivary aMMP‐8 showed significant strong positive correlations with all periodontal parameters (*p* < 0.01) except a moderate correlation with FMPS. Total MMP‐8 weakly and non‐significantly correlated with BoP, FMPS, and CAL. Salivary MPO demonstrated significant moderate positive correlations with all periodontal parameters (*p* < 0.01) (Data S1).

### 3.4 Correlation of systemic parameters with aMMP‐8, tMMP‐8, and MPO

WC exhibited a moderate positive correlation with aMMP‐8 (*r* = 0.329, *p* < 0.001) and MPO (*r* = 0.304, *p* = 0.001) but not with tMMP‐8. Systolic and diastolic BP demonstrated weak positive correlations with aMMP‐8 and stronger correlations with MPO (*r* = 0.448, *p* < 0.001) versus non‐significant associations with tMMP‐8.

Serum TG showed a moderate positive correlation with aMMP‐8 (*r* = 0.283, *p* = 0.002) and MPO (*r* = 0.335, *p* < 0.001), but not with tMMP‐8 (*r* = −0.071, *p* > 0.05). aMMP‐8 also correlated positively with LDL (*r* = 0.428, *p* < 0.001), FBS (*r* = 0.371, *p* < 0.001), and HbA1c (*r* = 0.291, *p* < 0.001), while MPO correlations were non‐significant. tMMP‐8 exhibited weak inverse correlations with FBS (*r* = −0.220, *p* = 0.021) and HbA1c (*r* = −0.211, *p* = 0.021) (Data S1).

### 3.5 Correlation among aMMP‐8, tMMP‐8, and MPO

A significant moderate positive correlation was observed between salivary aMMP‐8 and tMMP‐8 levels (*r* = 0.318, *p* < 0.001). A similar correlation occurred between aMMP‐8 and MPO (*r* = 0.319, *p* < 0.001). However, salivary tMMP‐8 and MPO levels did not significantly correlate (Data S1).

## 4 DISCUSSION

The complex interaction of host, microbial, and environmental variables shapes the multifactorial pathogenesis of periodontitis and ultimately dictates disease progression. Considerable research interest has focused on the bidirectional and interlinked relationship between metabolic syndrome and periodontitis since both conditions relate to systemic inflammation and insulin resistance. Previous evidence indicates a positive association between metabolic syndrome and periodontal disease progression in middle‐aged individuals, likely due to an array of contributing systemic disorders (Dietrich et al., 2013; Jaramillo et al., 2017; Pirih et al., 2021).

This study aimed to investigate differences in periodontal and salivary parameters among participants aged 25–55 years categorized as those with metabolic syndrome and periodontal disease (MetS‐PD), systemically healthy with periodontal disease (SH‐PD), and systemically healthy with periodontal health (SH‐PH). Metabolic syndrome diagnosis in this study followed the 2006 IDF criteria (Rezaianzadeh et al., 2012).

Unlike previous studies that reported the effect of MetS on periodontitis patients irrespective of a specific severity, the present study focused on including participants based on the presence of interdental or buccal CAL ≥5 mm and PPD ≥6 mm in more than 30% of the teeth (Tonetti et al., 2018). We employed participants with stage 3 and 4 periodontitis for enhanced interpretation of associations between developing subclinical atherosclerosis, metabolic syndrome, or other systemic comorbidities and periodontitis (Nascimento et al., 2019).

MetS and periodontitis are attributed to shared inflammatory pathways, dysregulated adipokine production, oxidative stress, and matrix remodeling processes. These factors relate local periodontal inflammation to systemic metabolic abnormalities and cardiovascular consequences (Khosravi et al., 2013). Full‐mouth clinical periodontal assessment supplemented biochemical analysis of salivary biomarkers like aMMP‐8, tMMP‐8, and MPO to provide insight into disease pathogenesis. Participant distribution was homogeneous across confounding variables like age, sex, residence, education level, and oral health behavioral methods. Evidence reports on the Japanese elderly population indicate that MetS had a 2.6‐fold increased risk of developing periodontal disease, contributing to disease chronicity (Iwasaki et al., 2015). The inclusion of the elderly population in previous investigations may have contributed to the inconsistent results on brushing habits and the prevalence of metabolic syndrome (Kobayashi et al., 2012).

The current study demonstrated higher systemic and periodontal parameters, among periodontitis participants with MetS compared to those without MetS (all *p* < 0.01). These findings agree with Jung et al. (2023) regarding MetS components like central obesity, dyslipidemia, hypertension, hyperglycemia, and periodontal disease. Similarly, various other studies also report an increased prevalence of ≥5 mm PPD, ≥40% alveolar bone loss, ≥0.5 mm mobility, and tooth loss in metabolic syndrome (Campos et al., 2020; Kaye et al., 2016; Morita et al., 2009; Tegelberg et al., 2019). Our observation of more missing teeth in MetS‐PD aligns with previous evidence of higher metabolic syndrome risks in periodontitis subjects with <28 remaining teeth (Kawashita et al., 2017).

The elevated LDL, FBS, TG, and BP in MetS‐periodontitis participants resemble findings by Koo and Hong (2018). Moderate positive correlations between WC, BP, LDL, FBS, and all periodontal parameters agree with the findings of Kotin et al. (2022), implying an association between periodontal disease severity and central adiposity. HDL‐c did not significantly differ between groups, possibly reflecting the possibility of the effect of dietary intake of saturated fat (Yanai & Tada, 2018). Most periodontal parameters did not significantly correlate with HDL levels, similar to Pietropaoli et al. (2023), who found no HDL‐c linkage with periodontitis despite correlations between gingival bleeding and metabolic syndrome criteria. Research also indicates gender‐specific differences, with periodontitis severity in males correlating to low HDL and hypertriglyceridemia, while abdominal obesity and low HDL are associated more in women (Kim et al., 2019). Significant positive correlations between TG and periodontal parameters observed in the study signify the impact of lipid metabolism on periodontal health. This study aligns with previous evidence of increased frequency, severity, and extent of periodontitis among those with MetS (Campos et al., 2020; Jung et al., 2023).

Researchers have explored salivary biomarkers as sentinel molecules to differentiate healthy and diseased conditions. Compared to other biological fluids, saliva sampling has several advantages of being simple and non‐invasive, requiring minimum expertise. MMP‐8 contributes to vascular remodeling, atherosclerosis, and plaque instability, linking MetS to periodontal tissue destruction and systemic complications (Hopps & Caimi, 2012). MMP‐8, MMP‐9, and MMP‐13 in GCF were independently related to the coexistence of periodontitis and MetS (Han, Lim, et al., 2012). Moreover, observation by Katsiki P et al. on greater MMP‐8 in the saliva, mouth rinse, and GCF of periodontitis patients compared to control indicate the potential use of saliva as a surrogate biological fluid for the biomarker assessment instead of GCF (Katsiki et al., 2021).

Quantitative salivary analysis in this study revealed higher aMMP‐8 levels in MetS‐PD versus SH‐PD, with the lowest concentration in SH‐PH (*p* < 0.01). Elevated aMMP‐8 indicates increased proteolytically active inflammatory processes, warranting further investigation into the specific MetS and aMMP‐8 connection. These results agree with studies demonstrating the link between elevated circulating MMP‐9, MMP‐8, and TIMP‐1 and increased cardiovascular risk markers like adhesion molecules and inflammatory mediators in metabolic syndrome (Goncalves et al., 2009).

The high rate of positive qualitative real‐time aMMP‐8 point‐of‐care (POC) immunoassay results in MetS‐PD underscores a potential metabolic syndrome‐periodontal disease association. As Sorsa et al. (2022) note, aMMP‐8, but not total latent proMMP‐8, significantly mediates tissue degradation in periodontitis (Lee et al., 1995; Romanelli et al., 1999). The absence of positive tests in SH‐PH significantly contrasts with the other two diseased groups. These results are consistent with earlier research on the accuracy of aMMP‐8 POC tests in periodontitis screening, where false‐positive results are rarely detected (Deng et al., 2021).

Proinflammatory cytokines expressed in response to periodontal pathogens increase MPO secretion and mediate the progression of periodontal disease and cardiovascular risk (Monserrat‐Mesquida et al., 2020). Like aMMP‐8, salivary MPO significantly differed between groups (*p* < 0.01), with elevated MetS levels indicating heightened neutrophil presence and oxidative stress, potentially implicating more significant inflammation versus systemically healthy groups. These findings relate to reports of higher salivary and plasma MPO concentrations in chronic heart disease patients with/without periodontitis versus systemically healthy controls (Polizzi et al., 2020). Metabolic syndrome components may thus impact increased salivary MPO levels. Since the MetS group in the present study included participants taking medications for systemic conditions, controlled systemic parameters could also affect the expression of these salivary biomarkers.

The moderate positive correlations found between salivary aMMP‐8 (*r* = 0.329, *p* < 0.001), MPO (*r* = 0.304, *p* = 0.001), and WC further suggest the contribution of abdominal obesity in oral inflammation, unlike tMMP‐8, which showed no significant association (*p* > 0.05). MPO and its byproducts promote processes like foam cell formation, endothelial dysfunction, and MMP‐8 activation (Wang & Khalil, 2018). Compared to aMMP‐8 (*r* = 0.188, *p* = 0.040), MPO demonstrated a stronger correlation with BP (*r* = 0.448, *p* < 0.001). As Nijakowski et al. (2023) recent review highlights, salivary MPO is a promising diagnostic biomarker for systemic cardiovascular and gastrointestinal disorders irrespective of periodontal disease. The overall substantial positive aMMP‐8/MPO correlation (*r* = 0.319, *p* < 0.001) indicates a coordinated immunological proinflammatory, oxidative, and proteolytic response (Sorsa et al., 2006; Weiss, 1989).

In contrast to aMMP‐8, total MMP‐8 levels did not significantly differ between groups (*p* = 0.269), unlike Gupta et al. (2015), who found elevated salivary tMMP‐8 in T2DM patients with chronic periodontitis versus systemically healthy periodontitis and controls. The results further support the evidence that tMMP‐8 may not differentiate MetS or periodontitis stages (Räisänen et al., 2023). Our results resemble Collin et al.'s (2000) findings of similar salivary MMP activity/levels between T2DM and controls. Interpreting tMMP‐8 levels could introduce errors due to latent proMMP‐8 and aMMP8; aMMP‐8 better indicates disease activity (Räisänen et al., 2023; Sorsa et al., 2020). Noteworthy is that aMMP‐8 is not synonymous with tMMP‐8 in periodontal diagnostics (Räisänen et al., 2023; Sahni et al., 2024).

FBS and HbA1c positively and significantly correlated with aMMP‐8 (both *p* < 0.001) but not MPO, while salivary aMMP‐8 was also associated positively with TG and LDL (*p* = 0.002 and *p* < 0.001, respectively). These results agree with evidence linking serum MMP‐8 to age, FBS, BMI, TG, and TG/HDL ratio, suggesting salivary inflammatory biomarker relationships with glycemic control and metabolism as an expression of both products are controlled by the same gene (Aquilante et al., 2007; Hasty et al., 1990). The overall positive aMMP‐8/tMMP‐8 correlation (*r* = 0.318) indicates both markers respond to inflammation, though MPO does not directly correlate with tMMP‐8. Interestingly, MPO is one of the critical latent proMMP‐8 activators in inflammations with enhanced oxidative stress (Sorsa et al., 2006; Weiss, 1989).

## 5 LIMITATIONS

This study did not analyze clinical and systemic parameter differences by gender among the metabolic syndrome participants, preventing assessment of potential gender effects on the relationship between metabolic syndrome and periodontitis. While the age range spanned 25–55 years, the relatively small sample of younger adults within each group limited analyses stratified by age. Since we diagnosed periodontitis based on clinical observation alone, disease grading and incorporation of radiographic bone loss assessment in future studies could enable better analysis of the progression of metabolic syndrome. Individual factors like medications, smoking history, physical activity, and nutrition likely introduced confounding that we did not account for.

## 6 IMPLICATIONS AND FUTURE DIRECTIONS

These findings increase understanding of the complex interactions between metabolic, cardiovascular, and periodontal disease factors, enabling more targeted and personalized dental care approaches. The observed disparities in periodontal parameters and salivary biomarkers like aMMP‐8 and MPO reveal a potential bidirectional relationship between metabolic syndrome and oral health. Salivary diagnostics provides innovative approaches to disease identification, prevention, and management, and in the future expected to become an integral part of contemporary healthcare. In metabolic syndrome patients, chair‐side testing for biomarkers such as aMMP‐8 and MPO may facilitate earlier preventive detection and monitoring of periodontal disease progression. Future research should incorporate radiographic bone loss for disease grading, account for lifestyle factors, and analyze gender differences.

## 7 CONCLUSION

Research on the oral health‐MetS connection highlights the role of oral inflammation in developing hypertension, glucose intolerance, and dyslipidemia. Elevated salivary aMMP‐8 and MPO levels, alongside worse clinical periodontal parameters in MetS‐periodontitis participants compared to systemically healthy individuals, signify heightened neutrophil activity and oxidative stress in metabolic syndrome. In contrast, no notable tMMP‐8 differences between groups indicate that aMMP‐8 may better reflect periodontal disease severity. Collaboration between dental and medical professionals should emphasize maintaining good oral hygiene and healthy lifestyles. Furthermore, longitudinal research is warranted to determine causality and elucidate the complex interactions among metabolic syndrome, periodontal health, and salivary biomarkers.

## AUTHOR CONTRIBUTIONS

**Julie Toby Thomas:** Conceptualization; investigation; writing – original draft; project administration; validation. **Betsy Joseph:** Methodology; software; data curation; writing – review and editing. **Sajit Varghese:** Investigation; formal analysis; validation. **Nebu George Thomas:** Funding acquisition; visualization; resources. **Baiju Kamalasanan Vijayakumary:** Formal analysis; validation. **Timo Sorsa:** Writing – review and editing; resources; supervision. **Sukumaran Anil:** Supervision; resources; writing – review and editing. **Tuomas Waltimo:** Visualization; writing – review and editing; project administration.

## CONFLICT OF INTEREST STATEMENT

T. Sorsa is the inventor of the following patents relating to MMP‐8 lateral flow testing: US patents 5652223, 5736341, 5866432, 6143476, 2017/0023571A1 (granted 6.6.2019), WO2018/060553A1 (granted 31.5.2018), 10488415B2, and Japanese patent 2016‐554676. The other authors declare no conflicts of interest.

## INFORMED CONSENT

Written informed consent was obtained from all participants before study enrollment.

## CONSENT FOR PUBLICATION

Participants provided consent for the publication of anonymized study data.

## Supporting information


Data S1

## Data Availability

The data that support the findings of this study are available from the corresponding author upon reasonable request.

## PATIENT DEMOGRAPHICS, PERSONAL DETAILS, ORAL HYGIENE STATUS QUESTIONNAIRE

### 1

Questions to measure oral hygiene status: (Tick the appropriate answer)

**Table:**

1. How many times do you brush your teeth?	No brushing.	Once a day.	Twice a day or more.
2. How do you clean your teeth?	Toothbrush, fluoride toothpaste & dental floss.	Toothbrush, fluoride toothpaste.	Toothbrush only.
3. How often do you change your toothbrush?	Once in 3 months.	Once in 6 months or more.	
4. Do you use mouthwashes containing fluoride?	Often.	Sometimes.	Rare or never.
5. Do you complain of halitosis (bad smell from your mouth)?	Often.	Sometimes.	Rare or never.
6. Do you complain of bleeding on brushing or gingival bleeding?	Often.	Sometimes.	Rare or never.
7. How often do you visit the dental clinic for a check‐up?	Once a year or more often.	Once every few years or when there is pain.	
8. What procedures do you do the most?	Scaling	Restorations	Extraction
9. How often do you get your teeth cleaned by a dentist?	Rare or never	Once in a year	Twice in a year
