# Supplementary material for: Association between metabolic syndrome and salivary MMP‐8, myeloperoxidase in periodontitis
Source: Oral Dis. 2024 Jun 9;31(1):225–38. doi: 10.1111/odi.15014 (PMC11808168; doi:10.1111/odi.15014)
Supplement: Supplementary file 1 — Data S1 [file ODI-31-225-s001.docx]

**Table 5.** Correlation Coefficients Between Periodontal Parameters and Metabolic Markers

| **Parameters**  ***(N=120)*** | **WC**  **(cm)** | **SBP**  **(mm Hg)** | **DBP**  **(mm Hg)** | **TG**  **(mg/dL)** | **LDL (mg/dL)** | **HDL (mg/dL)** | **FBS (mg/dL)** | **aMMP-8 (ng/mL)** | **tMMP-8 (ng/mL)** | **MPO**  **(ng/mL)** |
| --- | --- | --- | --- | --- | --- | --- | --- | --- | --- | --- |
| BOP (%) | 0.326^**^ | 0.269^**^ | 0.313^**^ | 0.329^**^ | 0.221^*^ | -0.024 | 0.342^**^ | 0.601^**^ | -0.052 | 0.323^**^ |
| FMPS (%) | 0.180^*^ | 0.116 | 0.257^**^ | 0.187^*^ | 0.241^**^ | 0.123 | 0.141 | 0.317^**^ | -0.034 | 0.296^**^ |
| PPD (mm) | 0.438^**^ | 0.325^**^ | 0.296^**^ | 0.342^**^ | 0.391^**^ | 0.090 | 0.422^**^ | 0.705^**^ | 0.010 | 0.351^**^ |
| GR (mm) | 0.353^**^ | 0.338^**^ | 0.282^**^ | 0.439^**^ | 0.343^**^ | -0.026 | 0.464^**^ | 0.570^**^ | 0.003 | 0.281^**^ |
| CAL (mm) | 0.483^**^ | 0.273^**^ | 0.256^**^ | 0.374^**^ | 0.420^**^ | 0.035 | 0.437^**^ | 0.776^**^ | -0.024 | 0.363^**^ |
| Number of missing teeth | 0.418^**^ | 0.350^**^ | 0.439^**^ | 0.175 NS | 0.311^**^ | 0.004 | 0.321^**^ | 0.550^**^ | 0.551^**^ | 0.284^**^ |

**Statistically significant at 1% level (*p*<0.01) ; *Significant at 5% level (*p*<0.05)

*Abbreviations:* MetS, Metabolic syndrome; WC,Waist circumference; SBP,Systolic blood pressure; DBP,Diastolic blood pressure; TG, Triglycerides; LDL,Low-density lipoprotein; HDL,High-density lipoprotein; FBS, Fasting blood sugar; BOP, Bleeding on probing; FMPS,Full mouth plaque score; PPD,Probing pocket depth; GR,Gingival recession; CAL,Clinical attachment loss

**Table 6.** Correlation Coefficients Between Metabolic Markers and Salivary Biomarkers

| **Parameters (*N=120)*** | **aMMP-8 (ng/ml)** | **tMMP-8 (ng/ml)** | **MPO (ng/ml)** |
| --- | --- | --- | --- |
| WC (cm) | 0.329^**^ | -0.152 | 0.304^**^ |
| SBP (mm of Hg) | 0.188^*^ | 0.029 | 0.351^**^ |
| DBP (mm of Hg) | 0.188^*^ | 0.022 | 0.448^**^ |
| TG (mg/dl) | 0.283^**^ | -0.071 | 0.335^**^ |
| LDL (mg/dl) | 0.428^**^ | 0.067 | 0.223^*^ |
| HDL (mg/dl) | 0.135 | 0.141 | 0.230^*^ |
| FBS (mg/dl) | 0.371^**^ | -0.220^*^ | 0.104 |
| HbA1c (%) | 0.291^**^ | -0.211^*^ | 0.064 |

**Statistically significant at 1% level (*p*<0.01); *Significant at 5% level (*p*<0.05)

*Abbreviations:* MetS,Metabolic syndrome; WC,Waist circumference; SBP,Systolic blood pressure; DBP,Diastolic blood pressure; TG .Triglycerides; LDL,Low-density lipoprotein; HDL,High-density lipoprotein; FBS, Fasting blood sugar; HbA1c, Glycated hemoglobin; aMMP,Active matrix metalloproteinase; tMMP,Total matrix metalloproteinase; MPO, Myeloperoxidase

**Table 7.** Correlation (r) among salivary expression of aMMP-8, tMMP-8, and MPO

| **Biochemical parameters**  ***(N=120)*** | **aMMP-8 (ng/ml)** | **tMMP-8 (ng/ml)** | **MPO (ng/ml)** |
| --- | --- | --- | --- |
| **aMMP-8 (ng/ml)** | 1 | 0.318^**^ | 0.319^**^ |
| **tMMP-8 (ng/ml)** | 0.318^**^ | 1 | 0.068 |
| **MPO (ng/ml)** | 0.319^**^ | .068 | 1 |

**Statistically significant at 1% level (*p*<0.01).

*Abbreviations:*aMMP,Active matrix metalloproteinase; tMMP,Total matrix metalloproteinase; MPO, Myeloperoxidase
